# Tryptophan modulates the impact of prolactin on insomnia in perimenopausal women: a cross-sectional study

**DOI:** 10.3389/fpsyt.2026.1809106

**Published:** 2026-05-01

**Authors:** Shizhuo Yang, Lanrong Sun, Weiyi Liu, Chaoyi Guo, Kexin Wang, Hong Huang, Peipei Zhou, Zhonglu Liao, Yanxiang Chang, Wenyan Wang, Yu-Hsin Chen, Xingguang Luo, Yimin Kang, Yanlong Liu, Sishi Du, Fan Wang, Qiulin Wu

**Affiliations:** 1 Department of Pharmacy, West China Tianfu Hospital, Sichuan University, Chengdu, China; 2 Department of Pharmacy, West China Hospital, Sichuan University, Chengdu, China; 3 School of Mental Health, Wenzhou Medical University, Wenzhou, China; 4 School of Public Health, Wenzhou Medical University, Wenzhou, China; 5 College of Pharmacy, Key Laboratory of Molecular Pharmacology and Drug Evaluation (Yantai University), Ministry of Education, Collaborative Innovation Center of Advanced Drug Delivery System and Biotech Drugs in Universities of Shandong, Yantai University, Yantai, China; 6 Department of Psychiatry, Yale University School of Medicine, New Haven, CT, United States; 7 Psychosomatic Medicine Research Division, Inner Mongolia Medical University, Huhhot, China; 8 Beijing Hui-Long-Guan Hospital, Peking University, Beijing, China; 9 Second Affiliated Hospital of Xinjiang Medical University, Urumqi, China; 10 Lishui Second People’s Hospital, Wenzhou Medical University, Lishui, China

**Keywords:** insomnia, perimenopausal, prolactin, sleep disorders, tryptophan

## Abstract

**Background:**

Insomnia is highly prevalent among perimenopausal women and exerts detrimental effects on physical health, psychological well-being, and overall quality of life. However, its underlying mechanisms remain incompletely understood. This cross-sectional study aimed to identify factors associated with insomnia in perimenopausal women.

**Methods:**

A total of 187 perimenopausal women aged 45–55 years were enrolled. Insomnia, anxiety, and depression severity were assessed using the Insomnia Severity Index (ISI), Generalized Anxiety Disorder-7 (GAD-7), and Patient Health Questionnaire-9 (PHQ-9), respectively. Serum levels of relevant amino acids and hormones were measured. Spearman correlation and linear regression analyses were performed to examine the associations among prolactin levels, tryptophan levels, insomnia, anxiety, and depression. Moderation analysis was further conducted to evaluate the potential moderating role of tryptophan in these relationships.

**Results:**

Serum prolactin levels were positively associated with scores of ISI, GAD-7, and PHQ-9. Furthermore, prolactin levels were positively correlated with the severity of sleep-onset difficulties, sleep maintenance problems, noticeability of impairment, and sleep-related distress. Of note, serum tryptophan levels significantly moderated the association between prolactin levels and ISI scores (*β* = 0.227, 95% CI = 0.04–0.41, *p* = 0.0148). To wit, he positive relationship between prolactin levels and insomnia severity was stronger in perimenopausal women with higher serum tryptophan levels compared with those with lower levels.

**Conclusions:**

The moderating effect of serum tryptophan on the relationship between prolactin levels and insomnia in perimenopausal women helps us understand the neuroendocrine mechanisms underlying perimenopausal insomnia and may inform future research on targeted preventive and therapeutic strategies.

## 1 Introduction

Perimenopause is a natural physiological transition preceding menopause, marked by a gradual decline in ovarian function and accompanied by pronounced fluctuations in gonadal hormones, including estrogen, progesterone, and testosterone (1, 2). Perimenopause encompasses a critical physiological transition characterized by menstrual irregularities and declining ovarian function, spanning from the onset of perimenopausal symptoms to one year following the final menstrual period. This phase typically occurs between the ages of 45 and 55 (3). During this period, approximately 80%–90% of women experience a range of perimenopausal and menopausal symptoms, such as insomnia, hot flashes, night sweats, palpitations, menstrual irregularities, anxiety, depression, and osteoporosis (4, 5). Among these manifestations, insomnia is one of the most prevalent and distressing features of perimenopausal syndrome (6). According to the *Diagnostic and Statistical Manual of Mental Disorders, Fifth Edition, Text Revision* (DSM-5-TR) (7), insomnia is defined as persistent dissatisfaction with sleep quantity or quality accompanied by significant daytime functional impairment. In perimenopausal women, this is typically manifested as poor sleep quality, short duration, difficulty initiating and maintaining sleep, and vivid dreaming. These sleep disturbances are often accompanied by somatic and psychological symptoms, including dizziness, chest tightness, palpitations, anxiety, depression, and irritability (8). The perimenopausal transition is widely recognized as a “window of vulnerability” for the emergence of affective disturbances. Approximately 41% of perimenopausal women report mental health challenges, with anxiety and depression being the most prevalent manifestations (9, 10). Emerging evidence underscores that women who develop mood disorders during this period often experience a more protracted disease course, a higher frequency of annual recurrences, and an elevated risk of suicidal ideation (11). Furthermore, affective disturbances serve as a robust predictor of sleep impairment, and a bidirectional relationship has been postulated between sleep disruption and depressive symptoms (12, 13). Despite its high prevalence and clinical burden, the pathogenesis of chronic insomnia in perimenopausal women remains incompletely understood. Emerging evidence suggests that perimenopausal insomnia is closely associated with psychological stress, neuroendocrine dysregulation, and hormone fluctuations.

Prolactin is a peptide hormone primarily synthesized and secreted by lactotroph cells in the anterior pituitary gland (14). Although prolactin is traditionally recognized for its role in lactation and reproductive physiology, growing evidence indicates that it also plays an important yet underappreciated role in sleep regulation (15). Prolactin can modulate neuroendocrine activity and anxiety-related responses and is proteolytically cleaved into vasoinhibins within the hypothalamus (16). Notably, circulating prolactin levels exhibit a distinct sleep-dependent rhythm, with concentrations increasing during sleep and declining during wakefulness (17). A retrospective study reported that 42.3% of hospitalized patients with comorbid mental disorders and sleep disturbances exhibited elevated prolactin levels, suggesting a potential association between hyperprolactinemia and excessive daytime sleepiness secondary to nocturnal insomnia (18). Consistently, Miao et al. observed significantly higher prolactin levels in patients with insomnia compared with individuals without sleep disorders (19).

Tryptophan is an essential amino acid required for normal somatic growth and serves as a precursor for several biologically active metabolites, including nicotinamide, serotonin, melatonin, and kynurenine (20). Tryptophan crosses the blood–brain barrier (BBB) via a shared active transport system that also facilitates the entry of other large neutral amino acids (LNAAs), such as leucine, isoleucine, tyrosine, phenylalanine, and valine, into the central nervous system. Accumulating evidence suggests that the consumption of tryptophan-rich foods (e.g., milk and tryptophan-enriched cereals) or tryptophan supplementation is associated with improved sleep quality (21–23). Tryptophan metabolism primarily proceeds through one of three main pathways: the kynurenine (Kyn) pathway, the 5-hydroxytryptamine (5-HT) pathway, and the indole pathway. Within the 5-HT pathway, tryptophan is converted into serotonin, which not only serves as a precursor for melatonin synthesis but also directly contributes to the regulation of sleep–wake cycles (24, 25). Neuronal inflammation and other factors may activate the Kyn pathway, resulting in metabolic disturbance. This not only reduces the production of sleep-beneficial 5-HT and melatonin, but also increases the generation of 3−hydroxykynurenine (3-HK) and quinolinic acid (QA), representative neurotoxic metabolites, thereby exacerbating insomnia (26, 27). In contrast, gut microbiota metabolites including tryptamine and indolokine A5 enhance melatonin synthesis by activating the aryl hydrocarbon receptor (AhR), thereby prolonging total sleep duration and shortening sleep latency in mice (28).

Despite growing interest in hormonal and metabolic influences on sleep, the biological mechanisms underlying insomnia in perimenopausal women remain insufficiently characterized. This cross-sectional study hypothesizes that insomnia in perimenopausal women may be associated with prolactin and tryptophan, aiming to elucidate specific underlying mechanisms through correlational statistical analysis and to provide new insights into the influencing factors of sleep disturbances in this population.

## 2 Materials and methods

### 2.1 Study design

This study adopted a cross-sectional design to investigate hematological parameters associated with insomnia in perimenopausal women and to explore potential underlying mechanisms. Participants completed face-to-face interviews conducted by trained researchers and a comprehensive set of standardized questionnaires assessing sociodemographic characteristics and relevant clinical factors. In parallel, laboratory test results and findings from specialized clinical examinations were collected and recorded for each participant. The study protocol was approved by the Urumqi Fourth People’s Hospital and was conducted in accordance with the principles of the Declaration of Helsinki. Written informed consent was obtained from all participants prior to enrollment. In accordance with the ethical approval, all personally identifiable information was removed, and each participant was assigned a unique numerical code to ensure anonymity during data management and analysis.

### 2.2 Paticipants

One hundred and eighty-seven perimenopausal women ages between 45–55 years (N = 187; age_mean_ = 49.01) were recruited from the gynecology outpatient and inpatient departments of hospitals in northern China. All participants were of Han Chinese ethnicity, and the majority had attained a junior high school level of education.

The inclusion criteria were as follows: (a) irregular menstrual cycles, defined as at least two instances within the previous 10 months of menstrual cycle variation ≥ 7 days, or amenorrhea lasting ≥ 2 menstrual cycles or ≥ 60 days with a total duration of amenorrhea < 1 year (29); (b) no history of infectious diseases or active viral or microbial infections; (c) intact uterus and ovaries; (d) no use of medications for alleviating perimenopausal symptoms within the preceding 3 months; and (e) ability to complete questionnaires independently, provision of written informed consent, and willingness to participate in the study.

The exclusion criteria included: (a) untreated unexplained vaginal bleeding; (b) premature ovarian insufficiency, organic ovarian diseases, bilateral oophorectomy, breast tumors, or other painful conditions; (c) a history of sleep disorders prior to the onset of perimenopause; (d) thyroid disease or other endocrine disorders; (e) use of sex hormone preparations or participation in other clinical trials within the previous month; (f) obstructive sleep apnea syndrome; (g) substance dependence, including alcohol, nicotine, cocaine, or other illicit drugs; (h) severe comorbid conditions, such as cardiovascular or cerebrovascular disease, liver or kidney disease, diabetes mellitus, hematological disorders, or severe neuropsychiatric disorders; (i) impaired communication ability due to hearing impairment, intellectual disability, or language disorders; (j) a self-reported or clinical diagnosis of bipolar disorder or prolactinoma, or use of medications known to affect prolactin secretion.

### 2.3 Assessment of insomnia severity using the insomnia severity index

The Insomnia Severity Index (ISI) was used to evaluate sleep quality and insomnia severity among participants. The ISI is a validated 7-item self-report questionnaire that assesses multiple dimensions of insomnia, including difficulty with sleep initiation and maintenance (nocturnal and early morning awakenings), satisfaction with current sleep patterns, interference with daytime functioning, the perceived noticeability of sleep-related impairment, and the degree of distress or concern caused by sleep difficulties (30). Each item is rated on a 5-point Likert scale ranging from 0 to 4, yielding a total score between 0 and 28. Based on the total score, insomnia severity is categorized as no clinically significant insomnia (0–7), subthreshold insomnia (8–14), moderate clinical insomnia (15–21), or severe clinical insomnia (22–28) (31). All assessments were administered during the baseline visit by uniformly trained investigators using structured interviews.

### 2.4 Assessment of depressive and anxiety symptoms

Depressive and anxiety symptoms were assessed using the Patient Health Questionnaire-9 (PHQ-9) and the Generalized Anxiety Disorder-7 (GAD-7) scales, respectively. The PHQ-9 consists of nine items, each rated on a 4-point Likert scale (0 = not at all, 1 = several days, 2 = more than half the days, 3 = nearly every day), yielding a total score ranging from 0 to 27, with higher scores indicating greater severity of depressive symptoms. Based on established cutoffs, depression severity was classified as no clinically significant depression (0–4), mild depression (5–9), moderate depression (10–14), moderately severe depression (15–19), and severe depression (20–27) (32).

The GAD-7 comprises seven items scored on the same 4-point Likert scale, producing a total score ranging from 0 to 21, with higher scores reflecting greater anxiety severity. Anxiety severity was categorized as no clinically significant anxiety (0–4), mild anxiety (5–9), moderate anxiety (10–14), and severe anxiety (15–21) according to standard criteria (33). All assessments were administered at baseline by uniformly trained investigators using structured interviews.

### 2.5 Blood sample collection and laboratory measurements

Fasting venous blood samples (5 mL) were collected from all participants before 10:00 a.m. and transferred into gel-separating vacuum tubes. Blood sampling was performed via antecubital venipuncture. Samples were centrifuged at 4,000 rpm for 10 minutes using a low-speed centrifuge to obtain serum. Serum levels of tryptophan were measured by ultra-high-performance liquid chromatography–tandem mass spectrometry (UHPLC-MS/MS) using a QTRAP 6500+ system (Sciex, Framingham, MA, USA) equipped with an electrospray ionization (ESI) source operating in positive ion mode. Serum hormone concentrations were measured using an electrochemiluminescence immunoassay on a Cobas 8000 automatic biochemical analyzer. Remaining serum aliquots were stored at −80 °C for subsequent analyses.

The primary analytes examined in this study included tryptophan, prolactin, estradiol, progesterone, luteinizing hormone (LH), follicle-stimulating hormone (FSH), and testosterone.

### 2.6 Statistical analysis

All statistical analyses were conducted using R software (version 4.3.1), with statistical significance set at *p* < 0.05 (two-tailed). Based on ISI scores, participants were categorized into a non-insomnia group (ISI 0–7; n = 109) and an insomnia group (ISI 8–28; n = 78). Differences in demographic, lifestyle, and clinical characteristics between groups were compared accordingly.

Data distribution was assessed using the Shapiro–Wilk test, with the threshold for statistical significance set at α = 0.05 (**Supplementary Table 1**). Tryptophan concentrations were normally distributed in both the insomnia and non-insomnia groups. In contrast, all other variables—including age, education level, age at menarche, menstrual cycle length, hormone levels (estradiol, progesterone, luteinizing hormone, follicle-stimulating hormone, testosterone, and prolactin), ISI scores, GAD-7 scores, and PHQ-9 scores—deviated from normality in both groups. Accordingly, subsequent analyses were performed using non-parametric methods.

Continuous variables were compared using the Mann–Whitney U test and are presented as median (interquartile range, Q1–Q3). Categorical variables were analyzed using the chi-square test and are expressed as frequency (percentage). Associations among prolactin, tryptophan, insomnia severity (ISI), anxiety symptoms (GAD-7), and depressive symptoms (PHQ-9) were examined using Spearman’s rank correlation coefficients. The corresponding correlation coefficients (ρ) and *p*-values are reported in **Supplementary Table 2**.

After centering and standardizing the variables, separate linear regression models were constructed with prolactin as the independent variable and ISI, GAD-7, and PHQ-9 scores as dependent variables. Given the multidimensional nature of sleep, the associations between prolactin and individual components of sleep disturbance were further explored using both Spearman correlation and linear regression analyses. The effect sizes and corresponding *p*-values of the Spearman correlation analysis between prolactin levels and individual ISI sub-scores are presented in **Supplementary Table 3**.

To examine interaction effects, linear regression models were used to evaluate the relationship between the prolactin–tryptophan interaction term and ISI, GAD-7, and PHQ-9 scores. All regression models were adjusted for potential confounders, including age (years), education level (years), menstrual cycle length (days), age at menarche (years), and relevant hormone levels. In addition, stratified linear regression analyses were conducted to explore the association between prolactin and ISI scores across different tryptophan levels. Standardized regression coefficients (*β*) with their corresponding 95% confidence intervals (CI) and *p* values are presented for all models. Finally, a moderation conceptual model was developed, with tryptophan specified as the moderating variable.

## 3 Results

### 3.1 Sociodemographic and clinical characteristics

Perimenopausal women were categorized into a non-insomnia group and an insomnia group according to their ISI scores. As shown in **Table 1**, compared with women in the non-insomnia group, those in the insomnia group exhibited significantly higher PHQ-9 scores and greater depression severity, as well as higher GAD-7 scores and greater anxiety severity. No significant differences were observed between the two groups with respect to age, years of education, age at menarche, menstrual cycle length, serum levels of estradiol, progesterone, luteinizing hormone (LH), follicle-stimulating hormone (FSH), testosterone, prolactin, or tryptophan.

**Table 1:** Baseline characteristics and demographics of perimenopausal women grouped according to the ISI scores.

Variable	ISI scores		*P* value	Effect size
	Non-insomnia (ISI scores = 0-7, n = 109)	Insomnia (ISI scores = 8-28, n = 78)		
Age, years	48.0 (47.0, 51.0)	49.0 (47.0, 51.0)	0.851	0.014
Education, years	8.00 (8.00, 11.0)	8.00 (8.00, 15.0)	0.424	0.058
Age at menarche, years	15.0 (13.0, 16.0)	14.0 (13.0, 15.0)	0.257	0.083
Menstrual cycle length, days	30.0 (27.0, 30.0)	28.0 (28.0, 30.0)	0.241	0.086
Estradiol, ng/L	41.8 (20.4, 79.7)	51.2 (19.3, 79.8)	0.917	0.008
Progesterone, μg/L	0.320 (0.140, 0.550)	0.435 (0.170, 0.880)	0.171	0.100
Luteinizing hormone, IU/L	13.5 (6.38, 36.7)	16.7 (5.05, 37.6)	0.559	0.043
Follicle-stimulating hormone, IU/L	20.4 (8.80, 56.7)	21.0 (5.93, 51.3)	0.431	0.058
Testosterone, μg/L	0.200 (0.110, 0.290)	0.255 (0.130, 0.340)	0.052	0.142
Prolactin, μg/L	13.8 (10.2, 21.3)	15.4 (10.3, 27.4)	0.277	0.080
Tryptophan, μmol/L	44.1 (37.8, 49.0)	41.4 (37.8, 47.5)	0.208	0.092
PHQ-9 scores	1.00 (0, 4.00)	8.50 (4.25, 14.8)	<0.001	0.600
GAD-7 scores	1.00 (0, 3.00)	6.00 (2.00, 12.0)	<0.001	0.522
Depression severity			<0.001	0.519
0 (PHQ-9 scores = 0-4)	86.00 (81.13%)	20.00 (18.87%)		
1 (PHQ-9 scores = 5-27)	23.00 (28.40%)	58.00 (71.60%)		
Anxiety severity			<0.001	0.442
0 (GAD-7 scores = 0-4)	91.00 (74.60%)	31.00 (25.40%)		
1 (GAD-7 scores = 5-21)	18.00 (27.70%)	47.00 (72.30%)		

In the baseline comparison of subjects with ISI scores of 0–7 points and 8–28 points, all continuous data were analyzed using the Mann-Whitney U test, and categorical variables were reported using the Chi-square test. The effect size of Mann-Whitney U test is represented by *r* value. The effect size of Chi-square test is represented by Cramer V value.

### 3.2 Correlation analyses

**Figure 1:**
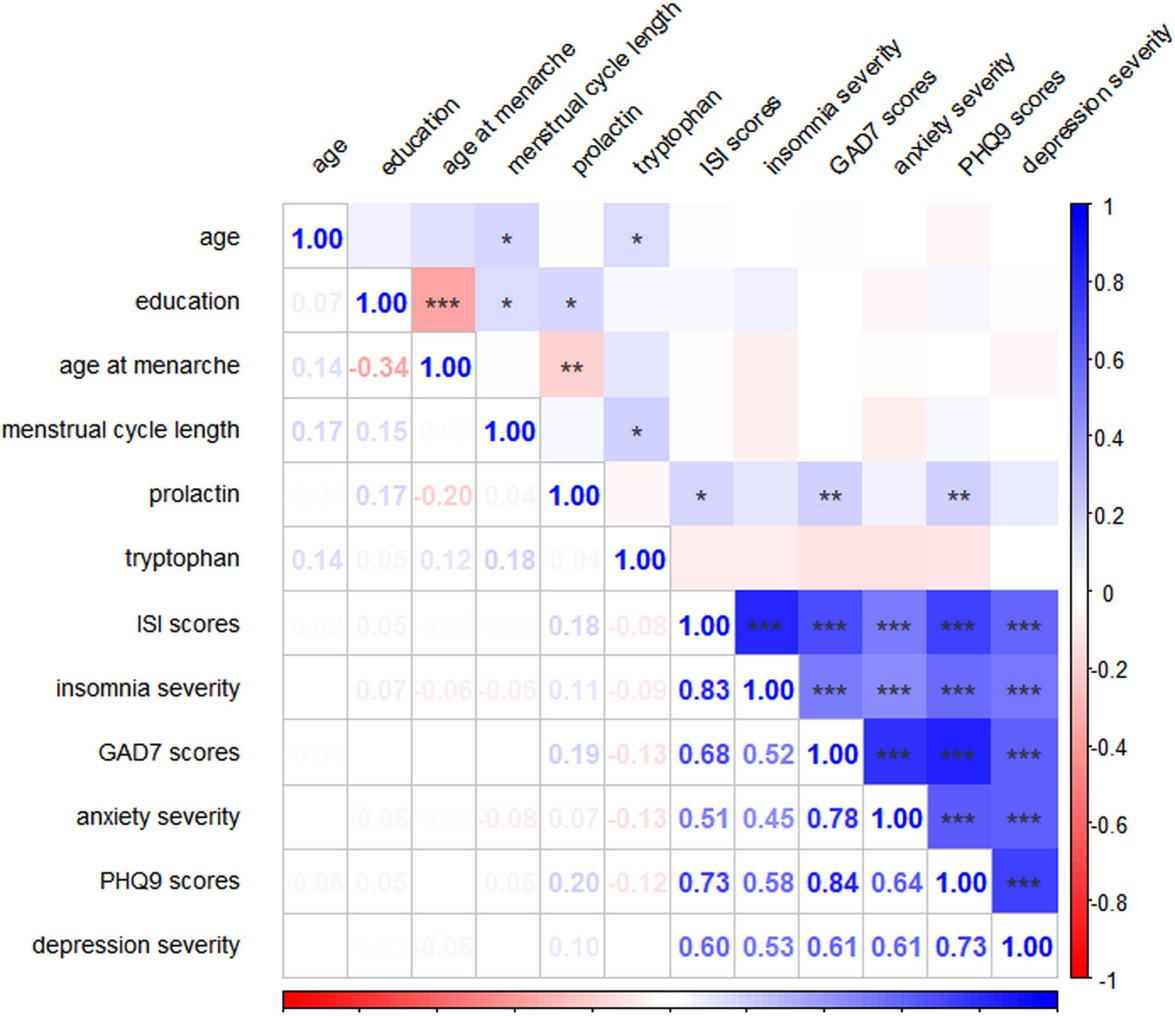
Correlation analysis between prolactin levels, tryptophan levels, ISI scores, insomnia severity, GAD-7 scores, anxiety severity, PHQ-9 scores and depression severity. Blue denotes positive correlation, red denotes negative correlation. Values indicate correlation coefficients (*r*). ^*^p < 0.05, ^**^p < 0.01, ^***^p < 0.001.

**Figure 2:**
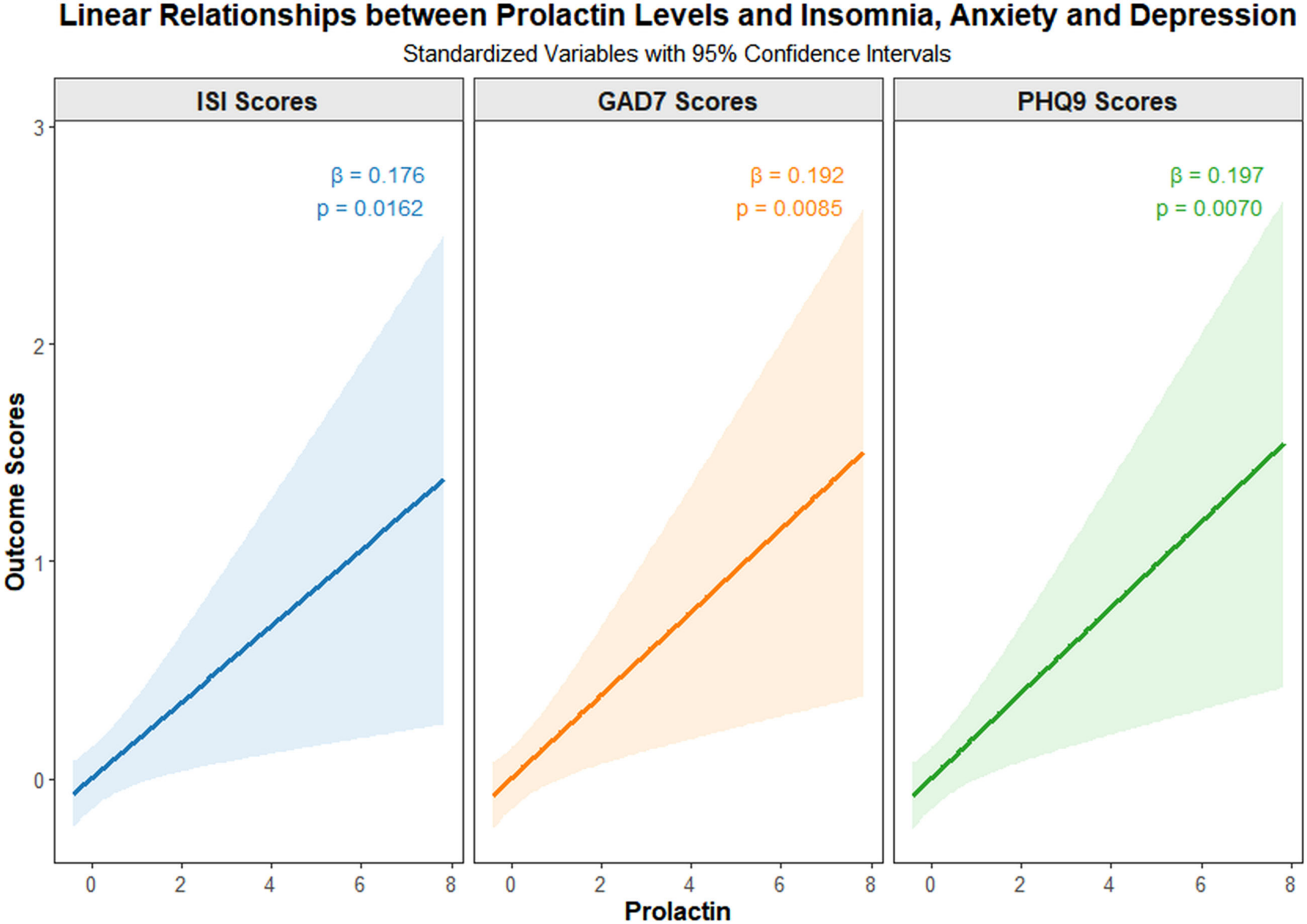
Linear regression analysis between prolactin, ISI scores, GAD-7 scores and PHQ-9 scores in perimenopausal women. *β* values represent an estimated effect size of the statistical analyses conducted.

Spearman correlation analyses revealed that age at menarche was significantly negatively associated with serum prolactin levels, *r (185*) = -0.20, *p* = 0.0065, while menstrual cycle length was significantly positively associated with serum tryptophan levels, *r (185*) = 0.18, *p* = 0.0134. In addition, prolactin levels showed significant positive correlations with ISI scores, *r (185*) = 0.18, *p* = 0.0162, GAD-7 scores, *r (185*) = 0.19, *p* = 0.0085, and PHQ-9 scores, *r (185*) = 0.20, *p* = 0.0070. Furthermore, insomnia severity was significantly positively correlated with both anxiety and depressive symptoms, as illustrated in **Figure 1**. The linear relationships between prolactin levels and ISI, GAD-7, and PHQ-9 scores are visualized in **Figure 2**. Specifically, ISI scores increased with higher prolactin levels, *β* = 0.176, *p* = 0.0162, as did GAD-7 scores, *β* = 0.192, *p* = 0.0085, and PHQ-9 scores, *β* = 0.197, *p* = 0.0070.

### 3.3 Associations between prolactin levels and specific sleep disturbance domains

**Figure 3:**
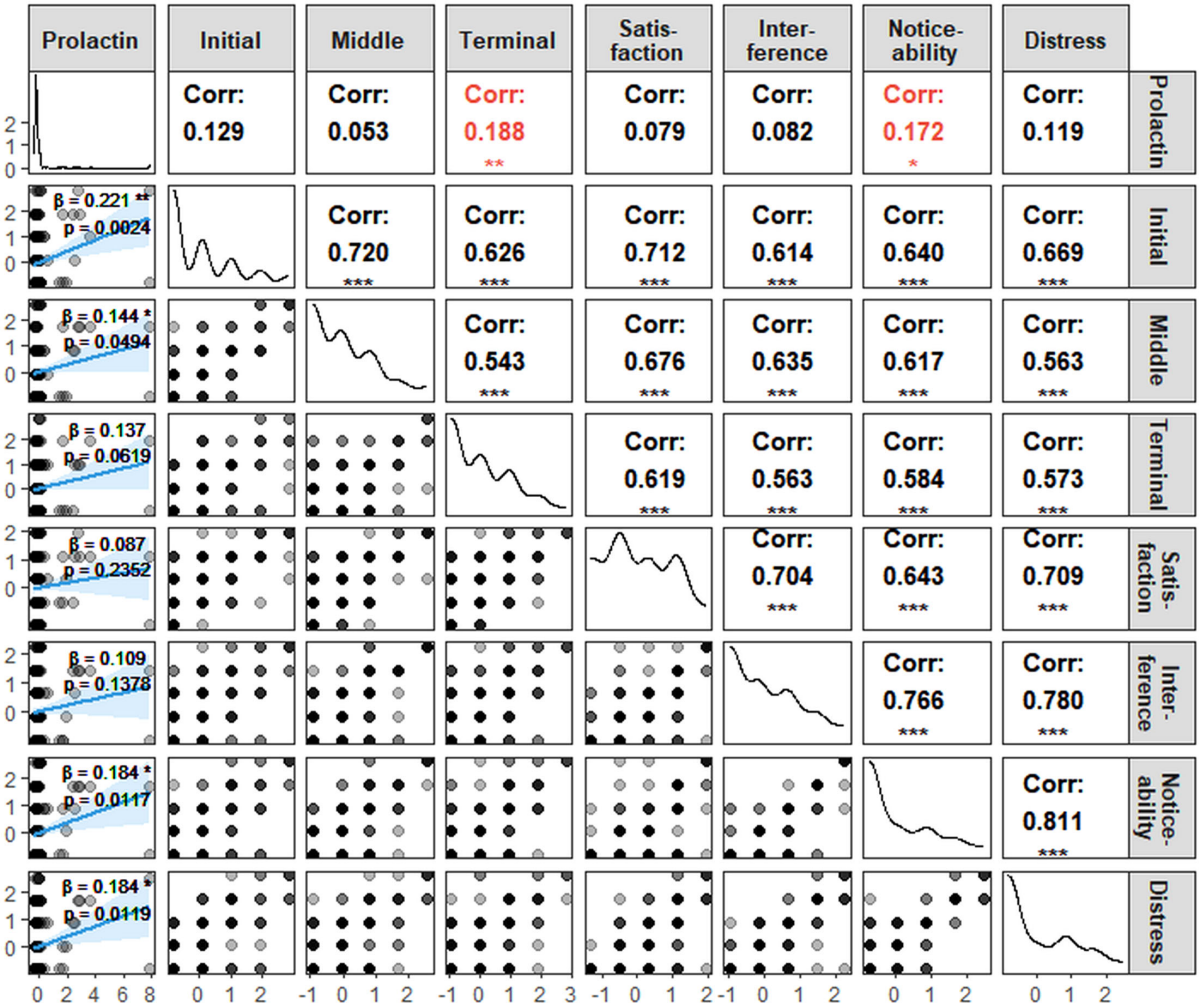
Associations of prolactin levels with specific sleep disturbance domains. Initial, sleep-onset difficulty; Middle, sleep maintenance problems; Teminal, early morning awakening; Satisfaction, satisfaction with current sleep patterns; Interference, interference with daytime functioning; Noticeability, noticeability of impairment; Distress, sleep-related distress. *β* values represent an estimated effect size of the statistical analyses conducted. ^*^p < 0.05, ^**^p < 0.01, ^***^p < 0.001.

The ISI comprises seven items assessing distinct dimensions of sleep disturbance, including sleep-onset difficulty, sleep maintenance problems, early morning awakening, satisfaction with current sleep pattern, interference with daily functioning, noticeability of sleep-related impairment, and sleep-related distress (30), as depicted in **Figure 3**. Associations between serum prolactin levels and individual ISI items were examined using both Spearman correlation and linear regression analyses. Spearman correlation analysis demonstrated that prolactin levels were significantly positively correlated with early morning awakening, *r (185*) = 0.188, *p* = 0.0098, and noticeability of impairment, *r (185*) = 0.172, *p* = 0.0185. Consistent with these findings, linear regression analyses revealed that higher prolactin levels were significantly associated with greater severity of sleep-onset difficulty (*β* = 0.221, *p* = 0.0024), sleep maintenance problems (*β* = 0.144, *p* = 0.0494), noticeability of impairment (*β* = 0.184, *p* = 0.0117), and sleep-related distress (*β* = 0.184, *p* = 0.0119).

### 3.4 Moderation analysis of tryptophan

**Figure 4:**
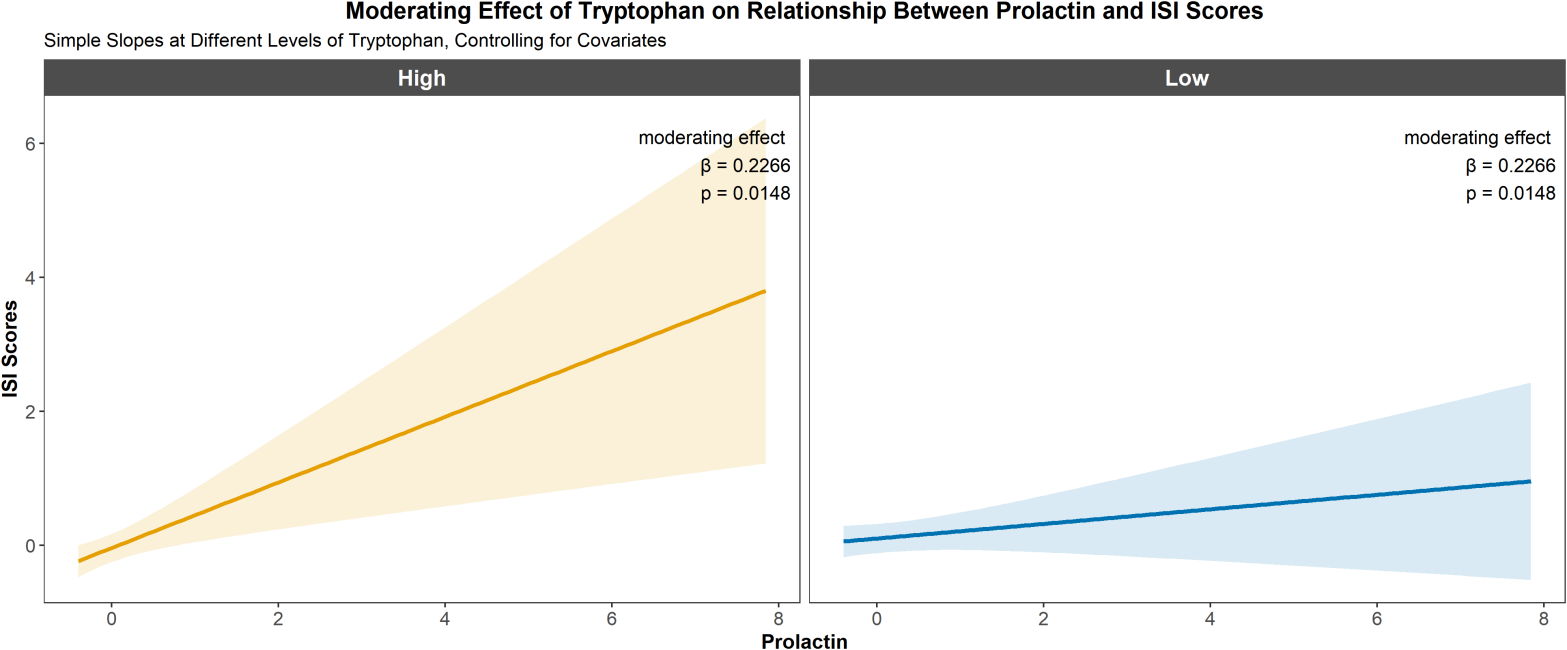
Linear regression analysis of prolactin levels versus ISI scores in populations with different tryptophan levels. *β* values represent an estimated effect size of the statistical analyses conducted.

**Figure 5:**
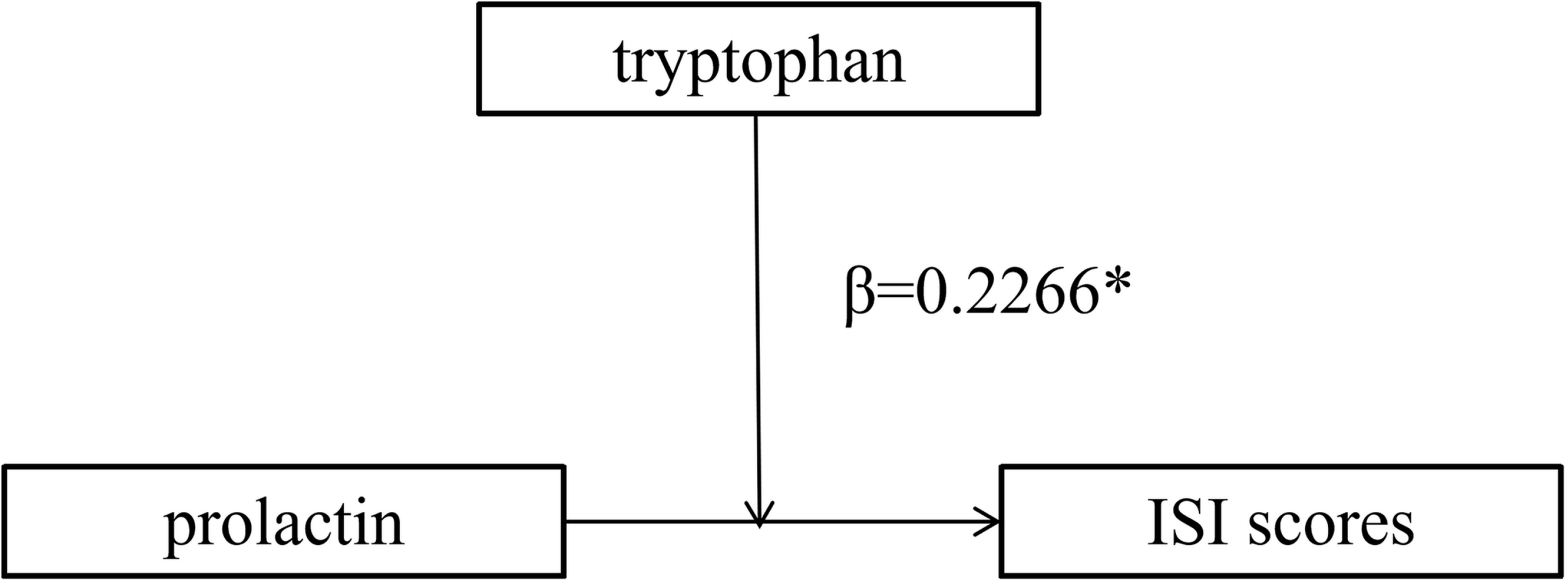
Model diagram of the moderating effect of tryptophan moderating the effect of prolactin and ISI scores in perimenopausal women. *β* values represent an estimated effect size of the statistical analyses conducted. *p < 0.05.

Based on the correlation results, moderation analyses were conducted to evaluate whether serum tryptophan levels modified the associations between prolactin levels and three clinical outcomes: ISI, GAD-7, and PHQ-9 scores. After adjusting for age, years of education, age at menarche, menstrual cycle length, and hormone levels, Model 1 revealed a significant positive association between prolactin levels and ISI scores (*β* = 0.191, 95% CI: 0.04–0.35, *p* = 0.0163; **Table 2**). In Model 2, no significant association was observed between tryptophan levels and ISI scores after adjusting for the same covariates. In Model 3, the prolactin × tryptophan interaction term was introduced to assess moderation. The results demonstrated a significant interaction effect, indicating that serum tryptophan levels moderated the association between prolactin levels and ISI scores (*β* = 0.227, 95% CI: 0.04–0.41, *p* = 0.0148). In contrast, no significant moderating effects of tryptophan were observed for the associations between prolactin levels and GAD-7 or PHQ-9 scores (see **Supplementary Tables 4**, **5**). To further elucidate the moderating effect, participants were stratified into high- and low-tryptophan groups based on serum tryptophan concentrations. Stratified analyses revealed that the positive association between prolactin levels and ISI scores was stronger among women with higher tryptophan levels compared with those with lower levels (**Figure 4**). The moderation effect of tryptophan on the relationship between prolactin levels and insomnia severity is illustrated in **Figure 5**.

**Table 2:** Linear regression table for the moderation analysis of tryptophan between prolactin levels and ISI scores.

Variable	Model 1				Model 2				Model 3			
	*β*	*t*	95% CI	*p*	*β*	*t*	95% CI	*p*	*β*	*t*	95%CI	*p*
Age	0.0572	0.740	(-0.1, 0.21)	0.4602	0.0535	0.681	(-0.10, 0.21)	0.4967	0.0497	0.647	(-0.10, 0.2)	0.5182
Education	0.0350	0.430	(-0.13, 0.20)	0.6674	0.0582	0.708	(-0.10, 0.22)	0.4797	0.0591	0.732	(-0.1, 0.22)	0.4651
Age at menarche	-0.0611	-0.074	(-0.17, 0.16)	0.9407	-0.0281	-0.341	(-0.19, 0.13)	0.7334	0.0248	0.303	(-0.14, 0.19)	0.7623
Menstrual cycle length	-0.0376	-0.494	(-0.19, 0.11)	0.6216	-0.0175	-0.226	(-0.17, 0.14)	0.8215	-0.0215	-0.283	(-0.17, 0.13)	0.7772
Estradiol	0.0029	0.035	(-0.16, 0.17)	0.9720	0.0136	0.162	(-0.15, 0.18)	0.8715	0.0021	0.026	(-0.16, 0.16)	0.9793
Progesterone	0.1341	1.770	(-0.02, 0.28)	0.0784	0.1405	1.833	(-0.01, 0.29)	0.0685	0.1303	1.745	(-0.02, 0.28)	0.0828
Luteinizing hormone	-0.0410	-0.361	(-0.28, 0.2)	0.7184	-0.0782	-0.636	(-0.32, 0.16)	0.5254	-0.0202	-0.168	(-0.26, 0.22)	0.8672
Follicle-stimulating hormone	-0.0288	-0.218	(-0.29, 0.23)	0.8278	0.0521	0.402	(-0.2, 0.31)	0.6881	-0.0570	-0.436	(-0.32, 0.2)	0.6630
Testosterone	-0.0108	-0.142	(-0.16, 0.14)	0.8873	-0.0327	-0.026	(-0.18, 0.12)	0.6703	-0.0087	-0.116	(-0.16, 0.14)	0.9079
Prolactin	0.1905^*^	2.426	(0.04, 0.35)	0.0163	–––	–––	––	––	0.3286^***^	3.408	(0.14, 0.52)	0.0007
Tryptophan	–––	–––	––	––	-0.0882	-1.156	(-0.24, 0.06)	0.2492	-0.0740	-0.995	(-0.22, 0.07)	0.6901
Prolactin: Tryptophan	–––	–––	––	––	–––	–––	––	––	0.2266^*^	2.461	(0.04, 0.41)	0.0148
R²	0.06043				0.03633				0.09804			
F	1.132 (10, 176)				0.664 (10, 176)				1.576 (12, 174)			

Model 1 was the linear regression model with prolactin levels as the independent variable and ISI scores as the dependent variable. Model 2 was the linear regression model with tryptophan levels as the independent variable and ISI scores as the dependent variable. Model 3 included both prolactin, tryptophan and prolactin × tryptophan as independent variables based on Model 1. All the models were adjusted for age, education, age at menarche, menstrual cycle length and hormones (including estradiol, progesterone, luteinizing hormone, follicle-stimulating hormone and testosterone). All data were used for moderation analysis. *β* values represent an estimated effect size of the statistical analyses conducted. ^*^p < 0.05, ^***^p < 0.001.

## 4 Discussion

In the present study, serum prolactin levels were significantly and positively associated with insomnia severity, anxiety, and depressive symptoms, as reflected by higher ISI, GAD-7, and PHQ-9 scores. Beyond these overall associations, prolactin levels were linked to specific dimensions of insomnia, particularly greater difficulty with sleep onset, increased noticeability of daytime impairment by others, and heightened subjective distress related to sleep problems. Notably, serum tryptophan moderated the relationship between prolactin levels and insomnia severity. Specifically, among perimenopausal women with higher tryptophan levels, elevated prolactin concentrations were associated with increased ISI scores, indicating more severe insomnia symptoms. In contrast, this association was not observed in women with lower tryptophan levels.

In support of the findings that elevated prolactin levels are positively associated with insomnia and anxiety and mood level in the present study, a previous study reported that women with hyperprolactinemia experienced impaired nocturnal sleep quality and increased fatigue (34). Similarly, patients with insomnia exhibited higher prolactin concentrations, whereas individuals with prolactinoma reported no sleep disturbances (19). Among patients with prolactinoma, comorbid psychiatric and sleep-related symptoms are common, with anxiety, depressive symptoms, and sleep disturbances reported in 59.10%, 28.98%, and 9.10% of cases, respectively. Of note, some studies have failed to identify a direct association between circulating prolactin levels and sleep disturbances in patients with prolactinoma (35). Sex-specific differences have also been observed, with females demonstrating greater prolactin elevations and more pronounced antidepressant effects compared with males (36). Taken together, the data from the present study and previous studies suggest a modulating effect of circulating prolactin on sleep disturbance and related mood disorders, although this effect may vary with physiological (sex) and pathological (prolactinoma) conditions. Indeed, prolactin is known to attenuate stress-induced neuroendocrine and anxiety responses through a complex and context-dependent role in sleep and affective regulation (37, 38).

The majority of previous research has indicated that tryptophan exerts sleep-promoting effects in both humans and animal models. For example, Chun Siong et al. reported that combined administration of mulberry leaf extract and tryptophan improved sleep quality and post-awakening mood in adults with sleep disturbances (39). Sutanto et al. demonstrated that tryptophan supplementation significantly reduced wake after sleep onset (WASO) (23), while Bravo et al. found that consumption of tryptophan-rich cereal grains was associated with improved nocturnal sleep (40). These beneficial effects have been attributed either to the intrinsic bioactivity of tryptophan itself or to its role as a precursor for serotonin and melatonin synthesis within the central nervous system (23, 41, 42). Importantly, 5-hydroxytryptophan (5-HTP), an intermediate metabolite in the tryptophan–serotonin pathway, is capable of crossing the BBB (43).

It is worth noting that cerebral uptake of tryptophan is not determined solely by absolute plasma tryptophan concentrations, but rather by the ratio of tryptophan to other large neutral amino acids (the Trp–LNAA ratio), which compete for transport across the BBB via shared carrier systems (41). Indeed, dietary interventions such as α-lactalbumin supplementation and carbohydrate-rich, protein-poor diets have been shown to increase the plasma Trp–LNAA ratio, thereby facilitating enhanced brain tryptophan uptake and serotonergic neurotransmission (41, 44). In the present study, the moderating effect of tryptophan on the prolactin–insomnia relationship was more pronounced at higher circulating tryptophan levels, a finding that appears to diverge from the generally accepted sleep-promoting role of tryptophan. This apparent discrepancy may be explained by the fact that serum tryptophan concentrations do not necessarily reflect the fraction of tryptophan available for transport across the BBB and subsequent central nervous system activity. Elevated peripheral tryptophan levels may paradoxically be associated with reduced central bioavailability, depending on competing LNAA concentrations and transporter dynamics. Moreover, the identified regulatory role of tryptophan offers a novel perspective on the complex neuroendocrine interactions underlying sleep disturbances in perimenopausal women and highlights the need for future studies incorporating measures of the Trp–LNAA ratio and central serotonergic activity.

During perimenopause, estrogen levels decline progressively. Reduced estrogen bioavailability impairs dopamine synthesis and secretion; dopamine normally exerts an inhibitory effect on prolactin release. Consequently, diminished dopaminergic tone may predispose perimenopausal women to dysregulated or elevated prolactin secretion (45–47). Supraphysiological prolactin levels have been shown to induce mood disturbances (16, 19). In parallel, hyperprolactinemia is frequently accompanied by somatic symptoms such as mastalgia and cephalgia, which can further disrupt sleep continuity and quality (48, 49). Moreover, elevated prolactin levels perturb the homeostasis of key neurotransmitter systems, including dopaminergic, serotonergic, and γ-aminobutyric acid (GABAergic) pathways, thereby contributing to sleep disturbances (50–52). Increased prolactin concentrations also suppress estrogen biosynthesis in females (53). This estrogen deficiency may impair hypothalamic thermoregulatory function, precipitating vasomotor symptoms such as hot flashes and night sweats that markedly compromise sleep quality (54).

In addition to these physiological changes, perimenopausal women often experience increasing psychosocial stressors related to family responsibilities, career demands, and broader societal pressures. The convergence of hormonal fluctuations and chronic life stressors may exacerbate psychological distress in this population, thereby increasing vulnerability to anxiety and depression (5, 55, 56). Sustained exposure to chronic stress can further dysregulate the hypothalamic–pituitary–adrenal (HPA) axis, resulting in prolonged cortisol elevation and heightened sympathetic nervous system activity. These neuroendocrine alterations are known to impair both sleep initiation and the maintenance of restorative deep sleep (57).

Several limitations of this study should be acknowledged. First, the sample size was relatively modest, and participants were recruited solely from hospitals in northern China, which may limit the generalizability of the findings. Future studies with larger, multicenter, and ethnically diverse cohorts are warranted to validate and extend these results. Second, all outcome measures were based on self-reported questionnaires, which are subject to recall and reporting biases and may not fully align with objective sleep assessments. The incorporation of objective sleep measurements, such as polysomnography or actigraphy, would strengthen the reliability of future investigations. Third, the cross-sectional design precludes any causal inference. Well-powered prospective and longitudinal studies are therefore needed to further delineate the causal pathways linking prolactin, tryptophan, serotonergic signaling, circulating hormones, and sleep disturbances in perimenopausal women. Fourth, although participants with a self-reported history of bipolar disorder, prolactinoma, or use of prolactin-altering medications were excluded, the absence of structured clinical interviews to definitively rule out bipolar spectrum disorders and atypical depression subtypes, coupled with reliance on verbal reports for medical history, may have introduced information inaccuracies and potential confounding. Notwithstanding these limitations, the present study provides novel preliminary evidence regarding the interrelationships among prolactin, tryptophan, and sleep disturbances in perimenopausal women, which may inform future mechanistic and clinical research.

## 5 Conclusion

In conclusion, this study demonstrates a positive association between serum prolactin levels and insomnia severity in perimenopausal women, on which serum tryptophan exerts a modulatory effect. These findings contribute to a better understanding of the neuroendocrine mechanisms underlying insomnia in perimenopausal women, offer novel insights into potential therapeutic strategies for this population, and lay the groundwork for future investigations into the specific molecular and biological pathways involved.

## Data Availability

The raw data supporting the conclusions of this article will be made available by the authors, without undue reservation.
