# Supplementary material for: Tryptophan modulates the impact of prolactin on insomnia in perimenopausal women: a cross-sectional study
Source: Front Psychiatry. 2026 May 1;17:1809106. doi: 10.3389/fpsyt.2026.1809106 (PMC13176195; doi:10.3389/fpsyt.2026.1809106)
Supplement: Supplementary file 1 [file Table1.docx]

Supplementary Material

**Supplementary Table 1.** Normality test of demographic information between groups based on ISI scores.

| **Variable** | non-insomnia (ISI scores = 0-7, n = 109) | | insomnia (ISI scores = 8-28, n = 78) | |
| --- | --- | --- | --- | --- |
|  | effect sizes: W value | | *P* value | |
| **Age, years** | 0.929 | 0.928 | <0.001 | <0.001 |
| **Education, years** | 0.890 | 0.899 | <0.001 | <0.001 |
| **Age at menarche, years** | 0.954 | 0.900 | <0.001 | <0.001 |
| **Menstrual cycle length, days** | 0.822 | 0.863 | <0.001 | <0.001 |
| **Estradiol, ng/L** | 0.724 | 0.617 | <0.001 | <0.001 |
| **Progesterone, μg/L** | 0.382 | 0.534 | <0.001 | <0.001 |
| **Luteinizing hormone, IU/L** | 0.881 | 0.878 | <0.001 | <0.001 |
| **Follicle-stimulating hormone, IU/L** | 0.844 | 0.863 | <0.001 | <0.001 |
| **Testosterone, μg/L** | 0.114 | 0.226 | <0.001 | <0.001 |
| **Prolactin,** **μg/L** | 0.227 | 0.403 | <0.001 | <0.001 |
| **Tryptophan, μmol/L** | 0.995 | 0.988 | 0.967 | 0.668 |
| **PHQ-9 scores** | 0.814 | 0.897 | <0.001 | <0.001 |
| **Depression severity** | 0.501 | 0.544 | <0.001 | <0.001 |
| **GAD-7 scores** | 0.785 | 0.899 | <0.001 | <0.001 |
| **Anxiety severity** | 0.447 | 0.621 | <0.001 | <0.001 |

Note: In the normality test of demographic information of subjects with ISI scores of 0-7 points and 8-28 points, all data were analyzed using the Shapiro-Wilk test.

**Supplementary table 2.** Correlations among prolactin, tryptophan, insomnia, anxiety and depression.

| **Rho**  **(Cohen's d)**  ***P*** | **age** | **education** | **age at menarche** | **menstrual cycle length** | **prolactin** | **tryptophan** | **ISI scores** | **Insomnia severity** | **GAD7 scores** | **Anxiety severity** | **PHQ9 scores** | **Depression severity** |
| --- | --- | --- | --- | --- | --- | --- | --- | --- | --- | --- | --- | --- |
| **age** | 1 .00^***^ | 0.3193 | 0.0561 | 0.0218 | 0.6058 | 0.0486 | 0.7357 | 0.8945 | 0.5939 | 0.8878 | 0.4320 | 0.8155 |
| **education** | 0.07  (0.14) | 1 .00^***^ | 0.0000 | 0.0437 | 0.0183 | 0.5199 | 0.4767 | 0.3283 | 0.8123 | 0.5294 | 0.5376 | 0.6805 |
| **age at menarche** | 0.14  (0.28) | -0.34^***^  (-0.72) | 1 .00^***^ | 0.6531 | 0.0065 | 0.1070 | 0.6627 | 0.3996 | 0.9059 | 0.7204 | 0.9057 | 0.4671 |
| **menstrual cycle length** | 0.17^*^  (0.35) | 0.15^*^  (0.30) | 0.03  (0.06) | 1 .00^***^ | 0.5513 | 0.0134 | 0.7411 | 0.3930 | 0.8369 | 0.2810 | 0.4867 | 0.9005 |
| **prolactin** | -0.04  (-0.08) | 0.17  (0.35) | -0.20^**^  (-0.41) | 0.04  (0.08) | 1 .00^***^ | 0.5681 | 0.0162 | 0.1384 | 0.0085 | 0.3693 | 0.0070 | 0.1819 |
| **tryptophan** | 0.14^*^  (0.28) | 0.05  (0.10) | 0.12  (0.24) | 0.18^*^  (0.37) | -0.04  (-0.08) | 1 .00^***^ | 0.2621 | 0.2190 | 0.0717 | 0.0815 | 0.1070 | 0.8781 |
| **ISI scores** | 0.02  (0.04) | 0.05  (0.10) | -0.03  (-0.06) | -0.02  (-0.04) | 0.20^*^  (0.41) | -0.08  (-0.16) | 1 .00^***^ | 0.0000 | 0.0000 | 0.0000 | 0.0000 | 0.0000 |
| **Insomnia severity** | 0.01  (0.02) | 0.07  (0.14) | -0.06  (-0.12) | -0.06  (-0.12) | 0.11  (0.22) | -0.09  (-0.18) | 0.83^***^  (2.98) | 1 .00^***^  (1.22) | 0.0000 | 0.0000 | 0.0000 | 0.0000 |
| **GAD7 scores** | -0.04  (-0.08) | 0.02  (0.04) | -0.01  (-0.02) | 0.02  (0.04) | 0.19^**^  (0.39) | -0.13  (-0.26) | 0.68^***^  (1.85) | 0.52^***^ | 1 .00^***^ | 0.0000 | 0.0000 | 0.0000 |
| **Anxiety severity** | 0.01  (0.02) | -0.05  (-0.10) | -0.03  (-0.06) | -0.08  (-0.16) | 0.07  (0.14) | -0.13  (-0.26) | 0.51^***^  (1.19) | 0.45^***^  (1.01) | 0.78^***^  (2.49) | 1.00^***^ | 0.0000 | 0.0000 |
| **PHQ9 scores** | -0.04  (-0.08) | 0.05  (0.10) | -0.01  (-0.02) | 0.05  (0.10) | 0.18^**^  (0.37) | -0.12  (-0.24) | 0.73^***^  (2.14) | 0.58^***^  (1.42) | 0.84^***^  (3.10) | 0.64^***^  (1.67) | 1 .00^***^ | 0.0000 |
| **Depression severity** | 0.02  (0.04) | 0.03  (0.06) | -0.05  (-0.10) | -0.01  (-0.02) | 0.10  (0.20) | -0.01  (-0.02) | 0.60^***^  (1.50) | 0.53^***^  (1.25) | 0.61^***^  (1.54) | 0.61^***^  (1.54) | 0.73^***^  (2.14) | 1 .00^***^ |

Note: All data were reported as Spearman correlation analysis. The lower-left table shows the Spearman correlation effect size Rho (Cohen's d), and the upper-right table shows the corresponding P values. ^*^ *p*<0.05, ^**^*p*<0.01, ^***^*p*<0.001.

**Supplementary table 3.** Correlations between prolactin and Insomnia Severity Index dimensions.

| **Rho**  **(Cohen's d)**  ***P*** | **prolactin** | **Initial** | **Middle** | **Terminal** | **Satisfaction** | **Interference** | **Notice-ability** | **Distress** |
| --- | --- | --- | --- | --- | --- | --- | --- | --- |
| **prolactin** | 1 .00^***^ | 0.0781 | 0.4709 | 0.0098 | 0.2807 | 0.2640 | 0.0185 | 0.1054 |
| **Initial** | 0.13 (0.26) | 1 .00^***^ | 0.0000 | 0.0000 | 0.0000 | 0.0000 | 0.0000 | 0.0000 |
| **Middle** | 0.05 (0.10) | 0.72^***^ (2.08) | 1 .00^***^ | 0.0000 | 0.0000 | 0.0000 | 0.0000 | 0.0000 |
| **Terminal** | 0.19^**^ (0.39) | 0.63^***^ (1.62) | 0.54^***^ (1.28) | 1 .00^***^ | 0.0000 | 0.0000 | 0.0000 | 0.0000 |
| **Satisfaction** | 0.08 (0.16) | 0.71^***^ (2.02) | 0.68^***^ (1.85) | 0.62^***^ (1.58) | 1 .00^***^ | 0.0000 | 0.0000 | 0.0000 |
| **Interference** | 0.08 (0.16) | 0.61^***^ (1.54) | 0.64^***^ (1.67) | 0.56^***^ (1.35) | 0.70^***^ (1.96) | 1 .00^***^ | 0.0000 | 0.0000 |
| **Notice-ability** | 0.17^*^ (0.35) | 0.64^***^ (1.67) | 0.62^***^ (1.58) | 0.58^***^ (1.42) | 0.64^***^ (1.67) | 0.77^***^ (2.41) | 1 .00^***^ | 0.0000 |
| **Distress** | 0.12 (0.24) | 0.67^***^ (1.81) | 0.56^***^ (1.35) | 0.57^***^ (1.39) | 0.71^***^ (2.02) | 0.78^***^ (2.49) | 0.81^***^ (2.76) | 1 .00^***^ |

Note: All data were reported as Spearman correlation analysis. The lower-left table shows the Spearman correlation effect size Rho (Cohen's d), and the upper-right table shows the corresponding P values. ^*^*p*<0.05, ^**^*p*<0.01, ^***^*p*<0.001.

**Supplementary Table 4.** Linear regression table for the moderation analysis of tryptophan between prolactin levels and GAD-7 scores.

|  | **Model1** | | | | **Model2** | | | | **Model3** | | | |
| --- | --- | --- | --- | --- | --- | --- | --- | --- | --- | --- | --- | --- |
|  | ***β*** | ***t*** | **95%CI** | ***p*** | ***β*** | ***t*** | **95%CI** | ***p*** | ***β*** | ***t*** | **95%CI** | ***p*** |
| **Age** | -0.0248 | -0.323 | (-0.18, 0.13) | 0.7473 | -0.0272 | -0.347 | (-0.18, 0.13) | 0.7288 | -0.0214 | -0.280 | (-0.17, 0.13) | 0.7800 |
| **Education** | -0.0588 | -0.073 | (-0.17, 0.15) | 0.9420 | 0.0257 | 0.313 | (-0.14, 0.19) | 0.7547 | 0.0140 | 0.174 | (-0.14, 0.17) | 0.8624 |
| **Age at menarche** | 0.0464 | 0.570 | (-0.11, 0.21) | 0.5694 | 0.0206 | -0.250 | (-0.14, 0.18) | 0.8026 | 0.0736 | 0.902 | (-0.09, 0.23) | 0.3682 |
| **Menstrual cycle length** | 0.0143 | 0.190 | (-0.13, 0.16) | 0.8493 | -0.0439 | -0.566 | (-0.11, 0.2) | 0.5723 | 0.0364 | 0.483 | (-0.11, 0.19) | 0.6297 |
| **Estradiol** | -0.0531 | -0.647 | (-0.22, 0.11) | 0.5183 | -0.0393 | -0.471 | (-0.2, 0.13) | 0.6382 | -0.0533 | -0.656 | (-0.21, 0.11) | 0.5127 |
| **Progesterone** | 0.0666 | 0.886 | (-0.08, 0.21) | 0.3769 | 0.0750 | 0.980 | (-0.08, 0.23) | 0.3282 | 0.0648 | 0.870 | (-0.08, 0.21) | 0.3856 |
| **Luteinizing hormone** | 0.1977 | 1.632 | (-0.04, 0.44) | 0.1044 | 0.2529 | 1.247 | (-0.09, 0.39) | 0.2142 | 0.2095 | 1.739 | (-0.03, 0.45) | 0.0839 |
| **Follicle-stimulating hormone** | -0.3007^*^ | -2.269 | (-0.56, -0.04) | 0.0229 | -0.1956 | -1.514 | (-0.45, 0.06) | 0.1319 | -0.3151^*^ | -2.417 | (-0.57, -0.06) | 0.0167 |
| **Testosterone** | 0.0187 | 0.248 | (-0.13, 0.17) | 0.8042 | -0.0102 | -0.134 | (-0.16, 0.14) | 0.8937 | 0.0175 | 0.235 | (-0.13, 0.17) | 0.8149 |
| **Prolactin** | 0.2466^**^ | 3.165 | (0.09, 0.4) | 0.0018 | --- | --- | -- | --- | 0.3269^***^ | 3.398 | (0.14, 0.52) | 0.0009 |
| **Tryptophan** | --- | --- | -- | -- | -0.1382 | -1.815 | (-0.29, 0.01) | 0.0712 | -0.1249 | -1.684 | (-0.27, 0.02) | 0.0501 |
| **Prolactin : Tryptophan** | --- | --- | -- | -- | --- | --- | -- | -- | 0.1368 | 1.490 | (-0.04, 0.32) | 0.0148 |
| **R²** | 0.07529 | | | | 0.04062 | | | | 0.1025 | | | |
| **F** | 1.433 (10, 176) | | | | 0.7452 (10, 176) | | | | 1.656 (12, 174) | | | |

Note: Model 1 was the linear regression model with prolactin levels as the independent variable and GAD-7 scores as the dependent variable. Model 2 was the linear regression model with tryptophan levels as the independent variable and GAD-7 scores as the dependent variable. Model 3 included both prolactin, tryptophan and prolactin × tryptophan as independent variables based on Model 1. All the models were adjusted for age, education, age at menarche, menstrual cycle length and hormones (including estradiol, progesterone, luteinizing hormone, follicle-stimulating hormone and testosterone). All data were used for moderation analysis. *β* values represent an estimated effect size of the statistical analyses conducted. ^*^*p* < 0.05, ^**^*p* < 0.01, ^***^*p* < 0.001.

**Supplementary Table 5.** Linear regression table for the moderation analysis of tryptophan between prolactin levels and PHQ-9 scores.

|  | **Model1** | | | | **Model2** | | | | **Model3** | | | |
| --- | --- | --- | --- | --- | --- | --- | --- | --- | --- | --- | --- | --- |
|  | ***β*** | ***t*** | **95%CI** | ***p*** | ***β*** | ***t*** | **95%CI** | ***p*** | ***β*** | ***t*** | **95%CI** | ***p*** |
| **Age** | -0.0433 | -0.567 | (-0.19, 0.11) | 0.5715 | -0.04692 | -0.600 | (-0.2, 0.11) | 0.5491 | -0.0402 | -0.527 | (-0.19, 0.11) | 0.5990 |
| **Education** | -0.0345 | -0.430 | (-0.12, 0.19) | 0.6676 | 0.0660 | 0.807 | (-0.1, 0.23) | 0.4207 | 0.0531 | 0.663 | (-0.11, 0.21) | 0.5085 |
| **Age at menarche** | 0.0573 | 0.707 | (-0.1, 0.22) | 0.4806 | 0.0297 | -0.362 | (-0.13, 0.1) | 0.7181 | 0.0827 | 1.017 | (-0.08, 0.24) | 0.3104 |
| **Menstrual cycle length** | 0.0466 | 0.622 | (-0.1, 0.19) | 0.5346 | 0.0751 | 0.970 | (-0.08, 0.23) | 0.3333 | 0.0674 | 0.894 | (-0.08, 0.22) | 0.3725 |
| **Estradiol** | -0.0938 | -1.147 | (-0.26, 0.07) | 0.2528 | -0.0797 | -0.956 | (-0.24, 0.08) | 0.3402 | -0.0940 | -1.158 | (-0.25, 0.07) | 0.2483 |
| **Progesterone** | 0.0298 | 0.399 | (-0.12, 0.18) | 0.6902 | 0.0384 | 0.503 | (-0.11, 0.19) | 0.6154 | 0.0282 | 0.379 | (-0.12, 0.17) | 0.7048 |
| **Luteinizing hormone** | 0.1401 | 1.162 | (-0.1, 0.38) | 0.2468 | 0.0947 | 0.773 | (-0.15, 0.34) | 0.4403 | 0.1512 | 1.259 | (-0.09, 0.3) | 0.2099 |
| **Follicle-stimulating hormone** | -0.3099^*^ | -2.377 | (-0.57, -0.05) | 0.0185 | -0.2029 | -1.573 | (-0.46, 0.05) | 0.1175 | -0.3235^*^ | -2.487 | (-0.58, -0.07) | 0.0138 |
| **Testosterone** | 0.0151 | 0.201 | (-0.13, 0.16) | 0.8409 | -0.0141 | -0.186 | (-0.16, 0.14) | 0.8530 | 0.0140 | 0.118 | (-0.13, 0.16) | 0.8513 |
| **Prolactin** | 0.2518^**^ | 3.245 | (0.1, 0.4) | 0.0014 | --- | --- | -- | --- | 0.3272^**^ | 3.411 | (0.14, 0.52) | 0.0007 |
| **Tryptophan** | --- | --- | -- | -- | -0.1298 | -1.709 | (-0.28, 0.02) | 0.0893 | -0.1166 | -1.576 | (-0.26, 0.03) | 0.2308 |
| **Prolactin : Tryptophan** | --- | --- | -- | -- | --- | --- | -- | -- | 0.1287 | 1.405 | (-0.05, 0.31) | 0.1619 |
| **R²** | 0.08325 | | | | 0.04426 | | | | 0.1071 | | | |
| **F** | 1.598 (10, 176) | | | | 0.815 (10, 176) | | | | 1.739 (12, 174) | | | |

Note: Model 1 was the linear regression model with prolactin levels as the independent variable and PHQ-9 scores as the dependent variable. Model 2 was the linear regression model with tryptophan levels as the independent variable and PHQ-9 scores as the dependent variable. Model 3 included both prolactin, tryptophan and prolactin × tryptophan as independent variables based on Model 1. All the models were adjusted for age, education, age at menarche, menstrual cycle length and hormones (including estradiol, progesterone, luteinizing hormone, follicle-stimulating hormone and testosterone). All data were used for moderation analysis. *β* values represent an estimated effect size of the statistical analyses conducted. ^*^*p* < 0.05, ^**^*p* < 0.01.

**Detail Methods of The Statistical analyses**

In accordance with the principles of statistical inference for independent samples, the Shapiro–Wilk test was performed to assess the normality of all continuous variables prior to analysis. Participants were first divided into two groups based on their Insomnia Severity Index (ISI) scores: non-insomnia (ISI scores = 0-7, n = 109) and insomnia (ISI scores = 8-28, n = 78). The threshold for statistical significance was set at α = 0.05, and effect sizes were reported for all analyses to address Type I error concerns. All statistical analyses were conducted using R software (version 4.3.1) (1). The procedures for normality testing were as follows(2):

1. Establish the test hypothesis and determine the test level

H0: The overall distribution of each demographic indicator in the non-insomnia group (or insomnia group) follows a normal distribution.

H1: The overall distribution of each demographic indicator in the non-insomnia group (or insomnia group) does not follow a normal distribution.

α = 0.05.

1. Calculate the statistic Uniform ranking: Calculate the normal distribution statistics for the two groups respectively. The sample observations were arranged from smallest to largest, and the S-W test statistic W value was calculated according to the formula or output by the R studio shapiro.test function.
2. Determine the P-value and make the inference conclusion According to the "Shapiro-Wilk Test Statistic W Critical Value Table". The corresponding critical value was found according to the sample size n and the significance level α. Alternatively, the P-value can calculated by the R studio shapiro.test function. If P > 0.05: Do not reject H0, and consider the group data to follow a normal distribution. If P < 0.05: Reject H0, accept H1, and consider the group data not to follow a normal distribution.

The normality test results are presented in Supplementary Table 1. With the exception of tryptophan, all other variables in both groups and the total sample deviated significantly from normality (p < 0.05). Accordingly, the Mann–Whitney U test was used for between-group comparisons of baseline characteristics, and demographic data are presented as median (first quartile, third quartile) in Table 1. Spearman’s rank correlation was uniformly applied in all correlation analyses to ensure methodological consistency and robustness. The procedures for the Mann–Whitney U test were as follows(2):

1. Establish the test hypothesis and determine the test level

H0: The overall distribution of each demographic indicator is the same between the non-insomnia group and the insomnia group.

H1: The overall distribution of each demographic indicator is different between the non-insomnia group and the insomnia group.

α = 0.05.

1. Calculate the statistic Uniform ranking: Mix the raw data of the two groups and arrange the sequence numbers as “ranks” uniformly from smallest to largest. If the values are the same, take the average rank. Calculate the rank sum: Calculate the rank sums T1 and T2 for the two groups respectively. Calculate the U value: Calculate according to the formula or use the wilcox.test in R studio to output the statistic U value.

3. Determine the P-value and make the inference conclusion Use the wilcox.test in R studio to output the P-value. If P > α: At the level of α = 0.05, do not reject H0, and it cannot yet be considered that the overall distributions of the two groups are different. If P < α: At the level of α = 0.05, reject H0, accept H1, and consider that the overall distributions of the two groups are different that the difference is statistically significant.

Spearman’s rank correlation was used to examine: 1) the associations among prolactin, tryptophan, insomnia, anxiety, and depression in perimenopausal women; and 2) the associations between prolactin and each ISI sub-dimension. The analytical procedures were as follows(2):

1. Establish the test hypothesis:

H0: The overall rank correlation coefficient Rho of the two variables is 0, there is no correlation between the two variables.

H1: The overall rank correlation coefficient Rho of the two variables is not 0, there is a correlation between the two variables.

α = 0.05.

1. Calculate the statistic Ranking: Arrange the raw observations of the two variables from smallest to largest respectively, and use the sequence numbers (ranks) instead of the raw values. If the values are the same, take the average rank. Calculate the rank difference d for each pair of observations. The correlation coefficient Rho was calculated according to the formula or using the cor.test function in R Studio, and then converted into Cohen's d to measure the effect size.
2. Determine the P-value and make the inference conclusion Use the Z-test for approximate inference or use the R studio rcorr.test to output the significance P-value. If P > α: At the level of α = 0.05, do not reject H0, and it cannot yet be considered that there is a correlation between the two variables. If P < α: At the level of α = 0.05, reject H0, accept H1, and consider that there is a correlation between the two variables that the difference is statistically significant.

Spearman correlation coefficients Rho(r value) and corresponding p-values are presented in Supplementary Tables 2 and 3. Detailed statistical descriptions have been incorporated into the revised manuscript.

Reference

1. Field, Andy P. 2026. Discovering Statistics Using R and RStudio. London: SAGE Publications.

2. LANG T A, ALTMAN D G. Basic statistical reporting for articles published in biomedical journals: the "Statistical Analyses and Methods in the Published Literature" or the SAMPL Guidelines [J]. *Int J Nurs Stud*, 2015, 52(1): 5-9.
